# Genetics: Is LADA just late onset type 1 diabetes?

**DOI:** 10.3389/fendo.2022.916698

**Published:** 2022-08-10

**Authors:** M. Hernández, Y. Nóvoa-Medina, R. Faner, E. Palou, A. Esquerda, E. Castelblanco, A. M. Wägner, D. Mauricio

**Affiliations:** ^1^Department of Endocrinology and Nutrition, University Hospital Arnau de Vilanova, Lleida, Spain; ^2^Lleida Biomedical Research Institute (IRB Lleida), University of Lleida (UdL), Lleida, Spain; ^3^Department of Pediatrics, Complejo Hospitalario Universitario Insular Materno-Infantil, Las Palmas de Gran Canaria, Spain; ^4^Research Institute in Biomedical and Health Sciences (IUIBS), University of Las Palmas de Gran Canaria (ULPGC), Las Palmas de Gran Canaria, Spain; ^5^Histocompatibility and Immunogenetics Laboratory, Blood and Tissue Bank, Barcelona, Spain; ^6^Department of Laboratory Medicine, University Hospital Arnau de Vilanova, Lleida, Spain; ^7^Diabetis en Atenció Primària - Catalunya (DAP-Cat) Group, Unitat de Suport a la Recerca Barcelona, Fundació Institut Universitari per a la recerca a l’Atenció Primària de Salut Jordi Gol i Gurina, Barcelona, Spain; ^8^Division of Endocrinology, Metabolism and Lipid Research, Washington University School of Medicine in St. Louis, St. Louis, MO, United States; ^9^Department of Endocrinology and Nutrition, Complejo Hospitalario Universitario Insular Materno-Infantil, Las Palmas de Gran Canaria, Spain; ^10^Department of Endocrinology, Hospital de la Santa Creu i Sant Pau & Institut d’Investigació Biomèdica (IIB) Sant Pau, Barcelona, Spain; ^11^Consorcio Centro de Investigación Biomédica en Red (CIBER) of Diabetes and Associated Metabolic Diseases, Instituto de Salud Carlos III, Barcelona, Spain; ^12^Faculty of Medicine, University of Vic & Central University of Catalonia, Vic, Spain

**Keywords:** LADA (latent autoimmune diabetes in adults), genetics, HLA class II, PTPN22, Type 1 diabetes mellitus, INS, age of onset

## Abstract

**Background:**

There is a controversy regarding Latent Autoimmune Diabetes in Adults (LADA) classification and whether it should be considered a slowly progressing form of type 1 (T1) diabetes (DM) or a distinct type of DM altogether.

**Methods:**

This cross-sectional study assessed major genes associated with T1DM (class II *HLA*, *PTPN22* [rs2476601] and *INS* [rs689]) in patients with LADA, as compared with participants with T1DM (stratified according to age of diagnosis before or after 30) and T2DM. HLA genotyping of the *DRB1*, *DQA1* and *DQB1* loci was performed by reverse PCR sequence-specific oligonucleotides. HLA haplotypes were assigned according to those most frequently described in the European population. *INS* and *PTPN22* SNPs were genotyped by real-time PCR.

**Results:**

A total of 578 participants were included: 248 with T1DM (70 diagnosed after the age of 30), 256 with T2DM and 74 with LADA. High risk HLA alleles were significantly more frequent in LADA than in T2DM, whereas the opposite was true for protective alleles. We found a lower frequency of the high-risk DRB1*04-DQB1*03:02-DQA1*03:01 haplotype in LADA (21.1%) than in the overall T1DM (34.7%) (p<0.05), whereas no differences were found between these groups for DRB1*03-DQB1*02:01-DQA1*05:01 or for protective alleles. Only 12% the overall T1DM group had no risk alleles vs 30% of LADA (p<0.0005). However, HLA allele distribution was similar in LADA and T1DM diagnosed after the age of 30. A total of 506 individuals (195 with T1DM [21 diagnosed after age 30] 253 with T2DM and 58 with LADA) were genotyped for the *PTPN22* and *INS* SNPs. The G/A genotype of the *PTPN22* rs2476601 was more frequent and the T/T genotype of the *INS* SNP rs689 was less frequent in T1DM compared to LADA. We did not find any significant differences in the frequency of the mentioned SNPs between LADA and T2DM, or between LADA and T1DM diagnosed after the age of 30.

**Conclusion:**

In this relatively small cross-sectional study, the genetic profile of subjects with LADA showed a similar T1DM-related risk allele distribution as in participants with T1DM diagnosed after the age of 30, but fewer risk alleles than those diagnosed before 30. Differences were present for HLA, as well as *PTPN22* and *INS* genes.

## Introduction

Latent Autoimmune Diabetes in Adults (LADA) is a slowly progressive autoimmune form of diabetes presenting in adults. There is still debate regarding its classification and whether it should be considered a slowly progressing form of type 1 (T1) diabetes mellitus (DM) or a distinct type of DM (1). A recent consensus statement from the American Diabetes Association and the European Association for the Study of Diabetes (EASD) defines age of onset (>30 years), presence of diabetes associated autoantibodies and absence of insulin requirements for at least 6 months after diagnosis as key diagnostic criteria for LADA (2). Also, they recommend an individualized approach that differs from the classical management of T1DM.

It has been shown that individuals with LADA have a distinct metabolic profile compared to patients with T1DM and type 2 (T2) DM, with higher C-peptide concentrations than subjects with T1DM and a lower frequency of metabolic syndrome than people with T2DM (3). In addition, there is some evidence that LADA is associated with a genetic background that is different from T1DM and T2DM, with an attenuated influence of key T1DM-associated HLA haplotypes compared to T1DM and an increased presence of *PTPN22*, *INS* and *SH2B3* polymorphisms, compared to T2DM (4).

The *PTPN22* gene encodes a protein tyrosine phosphatase, a negative regulator for T cell activation, and a down-regulator of the immune response. The first study on the association between *PTPN22* gene variants with T1DM was reported by Bottini et al (5) in 2004. Among the single nucleotide polymorphisms (SNPs) in the gene, rs2476601 has been linked to T1DM (4, 6). The *INS* gene encodes the (pro) insulin protein. Variants in the gene or its promotor region might affect its thymic expression, leading to decreased tolerance and a higher likelihood of developing an autoimmune response (7).

In the present study, we evaluated differences in the HLA region, as well as in *PTPN22* and *INS*, in patients diagnosed with LADA compared to patients diagnosed with T1DM and T2DM.

## Methods

### Design and setting

This is a cross-sectional study, performed in Catalonia, Spain, assessing the major genes associated with T1DM in patients with LADA, as compared with individuals with T1DM and T2DM. It was part of a more comprehensive study, characterizing LADA in our population (3, 8). The study was designed in 2006. *PTPN22* and *INS*, along with HLA, were selected because they were at that time among the genes described to have more weight in the development of T1DM in genetic association studies (9).

### Patients

We included patients diagnosed with different forms of DM and different age groups. T1DM and T2DM were diagnosed according to standard criteria (1). GAD-antibody screening was performed in all participants. If they were positive, they were not classified as T2DM. Participants with T1DM were classified according to an age of onset of below or above 30 years of age. Patients with DM (both T1DM and LADA) diagnosed after the age of 30 years of age were recruited from two hospital-based centers in northeastern Spain (Barcelona and Lleida). The patients with T1DM diagnosed before 30 years of age were those entered in the Catalonian T1DM registry between January 1, 1987 and December 31, 1988 (10). Cases of LADA were defined as patients aged 30–70 years at the time of diagnosis of diabetes who did not require insulin for at least 6 months after diagnosis, with glutamic acid decarboxylase (GAD) autoantibody (GADAb) or tyrosine phosphatase autoantibody (IA-2Ab) positivity, as described in previous studies (3). All patients were included, at least, 6 months after diagnosis. Most LADA patients were diagnosed clinically, and further confirmed by GAD-antibody measurement at the reference hospital. The rest were identified in primary care among people with a clinical diagnosis of T2DM, after systematic antibody measurement as part of a larger study (8).

Samples from controls for the genetic analysis of *PTPN22* and *INS* genes were obtained from umbilical cord blood samples from the Barcelona Cord Blood Bank in 2009.

### Genetics

Automated DNA extraction was performed using the Maxwell instrument (Promega, Madison, Wisconsin, USA).

HLA genotyping of the *DRB1*, *DQA1* and *DQB1* loci was performed by reverse PCR-SSO (sequence-specific oligonucleotides) using INNO-LiPA HLA typing kits (Innogenetics, Fujirebio Europe N.V., Ghent, Belgium). High-risk and protective HLA-DRB1 alleles were determined by low resolution (1-field) genotyping, while *DQB1* and *DQA1* loci were typified by 2-field high resolution genotyping. HLA haplotypes were assigned according to the most frequent haplotypes described in European populations (11).

*INS* and *PTPN22* SNPs were genotyped using an in-house technique by real-time PCR on a LightCycler 480 PCR system (Roche Diagnostics, Basel, Switzerland) using FRET probes. Primer and probe sequences are shown in **Table 1**. The −23 HphI polymorphism (rs689) was genotyped as a surrogate marker for the *INS*-VNTR, based on the almost complete linkage disequilibrium between them (12) (the −23 HphI A allele corresponds to VNTR class I and the −23 HphI T allele corresponds to VNTR class III). In *PTPN22*, the SNP Arg620Trp (rs2476601) was genotyped.

**Table 1:** Primers and probes used for *INS* and *PTPN22* genotyping.

Name	Sequence	5’ Mod.	3’ Mod.
rs689_F	CAGCAGGGAGGACGTGGC		
rs689_R	CCCCGCACACTAGGTAGAGA		
rs689_anchor	GCCTTCAGCCTGCCTCAGCCCT		6-FAM-Q
rs689s_sensor	CCTGTCACCCAGATCAC	Cy-5	Phos
rs2476601_F	CCTCAAACTCAAGGCTC		
rs2476601_R	CCTTTGGATTGTTCTAATTAAC		
rs2476601_anchor	AATCATTTATTGTGGTTGAGGAAGCT	Cy-5	Phos
rs2476601_sensor	ACTTCCTGTACGGACACCT		6-FAM-Q

### Statistical analysis

Sample size estimations were done for the T1DM group as a whole. Indeed, the study was designed to detect an absolute 13% difference between groups in the absence of high-risk haplotypes [expected to be 13% in the participants with T1DM, 26% in the LADA group and 40% in the T2DM group] with a 95% confidence. This led to an estimation of 80 participants with LADA, 218 with T2DM and 256 with T1DM.A descriptive analysis was performed using percentages for qualitative variables and mean (standard deviation [SD]) or median (inter-quartile range [IQR]) for continuous variables, according to whether their distribution was Gaussian or not, respectively. Groups were compared using the Student’s t test or the Mann-Whitney U test (pairwise comparisons) and ANOVA or Kruskal-Wallis tests for continuous variables and chi-squared and Fisher’s test, as appropriate, for percentages. Hardy-Weinberg Equilibrium was assessed for the SNPs in *PTPN22* and *INS* and comparisons were made using the dominant, codominant, recessive and additive models, if possible. Results were expressed as odds ratios and 95% confidence intervals (OR [95% CI]). Both unadjusted and (age and BMI) adjusted comparisons (using a general linear model) were performed. Results were considered statistically significant if p was below 0.05

The study was approved by the Ethics Committees of Hospital de la Santa Creu i Sant Pau, Barcelona and Hospital Universitari Arnau de Vilanova, Lleida.

## Results

A total of 578 participants were included: 248 with T1DM (178 diagnosed below 30 years of age and 70 above that age), 256 with T2DM and 74 with LADA. Their main features are displayed in **Table 2**.

**Table 2:** Characterization of the participants according to type of diabetes.

	T1DM<30	T1DM>30	LADA	T2DM
**N**	178	70	74	256
**Female sex [n (%)]**	91 (51.1)	32 (45.7)	28 (38.3)	98 (37.8)
**Age (years)**	29.5 (8.6)*	47.9 (12.3)*	54.2 (12.2)	55.9 (9.3)*
**Diabetes duration (years)**	13.3 (5.9)*	5.4 (5)	5.2 (6.6)	2.7 (2.3)*
**Waist circumference (cm)**	87.2 (9.9)*	90.5 (12.7)	94.3 (12.1)	101.7 (13.6)*
**Body Mass Index (Kg/m^2^)**	22.1 (3.6)	25.7 (5.9)	26.5 (4.2)	30 (5.6)

Continuous variables are expressed as mean (standard deviation) T1DM<30: type 1 diabetes diagnosed before the age of 30. T1DM>30: type 1 diabetes diagnosed after the age of 30. *p ≤ 0.004 compared with LADA. T1DM, Type 1 Diabetes Mellitus; LADA, Latent Autoimmune Diabetes of the Adult; T2DM, Type 2 Diabetes Mellitus.

### Characterization

Patients with T1DM were significantly younger compared to patients with T2DM and LADA. Patients diagnosed with LADA also had a longer time since diagnosis compared to patients diagnosed with T2DM and those diagnosed with T1DM before the age of 30. Anthropometrical measurements in the participants with LADA showed values that fell between T1DM and T2DM (see **Table 2**).

### *HLA* analysis

**Table 3** summarizes the results of HLA genotyping. In a first analysis, we compared the frequency of HLA risk alleles between patients diagnosed with LADA (n = 74) and with T1DM (n = 248). We found a lower frequency of the DRB1*04-DQB1*03:02- DQA*03:01 allele in LADA (21.1%) vs T1DM (34.7%) (p <0.05). No differences were found regarding the frequency of DRB1*03-DQB1*02:01-DQA1*05:01 between these groups. No difference was found regarding the frequency of the protective alleles (DRB1*15-DQA1*01:02-DQB1*06:02) either. The presence of both risk haplotypes in the same individual (DRB1*03-DQB1*02:01/DRB1*04-DQB1*03:02) tended to be more frequent in T1DM than in LADA (p = 0.06). The absence of risk alleles, however, was more frequent in LADA than in T1DM (30.3% vs 12%) (p <0.001).

**Table 3:** HLA allele and haplotype distribution among groups.

	T1DM<30		T1DM>30		T1DM (total)		T2DM		LADA		T1DM vs. LADA	T2DM vs. LADA	T1DM>30 vs. LADA
	N	%	N	%	N	%	N	%	N	%	p-value	p-value	p-value
DRB1*04	92	51.7%	33	45.2%	125	49.8%	71	27.8%	24	31.6%	0.005	0.527	0.087
DRB1*04-DQB1*03:02	83	46.6%	30	41.1%	113	45.0%	58	22.7%	22	28.9%	0.013	0.268	0.120
DRB1*04-DQB1*03:02-DQA1*03:01	65	36.5%	22	30.1%	87	34.7%	44	17.3%	16	21.1%	0.039	0.157	0.318
DRB1*03-DQB1*02:01-DQA1*05:01	119	66.9%	28	38.4%	147	58.6%	48	18.8%	37	48.7%	0.128	<0.001	0.204
DQA1*03:01-DQA1*05:01	34	19.1%	5	6.8%	39	15.5%	9	3.5%	5	6.6%	0.045	0.246	0.947
DRB1*03–DQB1*02:01/DRB1*04- DQB1*03:02	43	24.2%	7	9.6%	50	19.9%	13	5.1%	8	10.5%	0.060	0.088	0.849
DRB1*15	4	2.2%	4	5.5%	8	3.2%	54	21.2%	4	5.3%	0.399	0.001	0.953
DRB1*15–DQA1*01:02-DQB1*06:02	2	1.1%	2	2.7%	4	1.6%	46	18.0%	4	5.3%	0.088	0.006	0.681
No risk alleles**	11	6.2%	19	26.0%	30	12.0%	149	58.4%	23	30.3%	<0.001	<0.001	0.566

**: no DRB1*03 or DRB1*04 or DQB1*02:01 or DQB1*03:02;

T1DM, Type 1 Diabetes Mellitus; LADA, Latent Autoimmune Diabetes of the Adult; T2DM, Type 2 Diabetes Mellitus.

When comparing the LADA and T2DM groups, we found a higher frequency of the T1DM risk haplotype DRB1*03-DQB1*02:01-DQA1*05:01 in the former (48.7% vs 18.8%; p <0.001), as well as a decreased frequency of the protective haplotype DRB1*15-DQA1*01:02-DQB1*06:02 (5.3% vs 18%, p <0.01).

Finally, a third sub analysis comparing the HLA risk and protective alleles in patients diagnosed with LADA vs T1DM diagnosed after 30 years of age found no differences between both groups.

### *PTPN22* and *INS*

**Table 4** summarizes the results of the *PTPN22* and *INS* SNP genotyping. Regarding *PTPN22* (rs2476601), the minor allele (T) frequency was 0.0432 in controls and the genotype distribution did not significantly depart from the Hardy Weinberg equilibrium (chi-squared 0.002). When comparisons were made between subjects with LADA and other groups, the T allele was present more frequently in patients with T1DM (both overall and those diagnosed before 30 years of age), than in controls and patients with T2DM and LADA. We did not find any significant differences in genotype distribution between T2DM and LADA, nor between T1DM diagnosed after the age of 30 and LADA in any of the models assessed.

**Table 4:** Polymorphisms in *PTPN22* and *INS* gene analysis in samples from controls (umbilical cord) and patients diagnosed with T1DM before (T1DM<30) and after 30 years of age (T1DM>30), LADA and T2DM.

Gene	SNP	Umbilical Cord	T1DM<30*	T1DM>30	LADA	T2DM
	C/C	224 (91.4%)	127 (73.0%)	16 (76.2%)	50 (86.2%)	220 (87%)
***PTPN22***	C/T	21 (8.6%)	46 (26.4%)	5 (23.8%)	8 (13.8)	32 (12.6%)
	T/T	0 (0%)	1 (0.6%)	0 (0%)	0 (0%)	1 (0.4%)
	A/A	123 (50.2%)	115 (66.1%)	12 (57.1%)	32 (55.2%)	121(47.8%)
***INS***	A/T	100 (40.8%)	55 (31.6%)	8 (38.1%)	20 (34.5%)	106 (41.9%)
	T/T	22 (9.0%)	4 (2.3%)	1 (4.8%)	6 (10.3%)	26 (10.6%)

*distribution among genotypes is significantly different (p<0.05) from LADA.

SNP: single nucleotide polymorphism; PTPN22: Protein Tyrosine Phosphatase Non-Receptor Type 22 gene; INS: Insulin gene; T1DM: Type 1 Diabetes Mellitus; LADA: Latent Autoimmune Diabetes of the Adult; T2DM: Type 2 Diabetes Mellitus.

Regarding *INS* (rs689), the frequency of the minor allele (T) in controls was 0.294, and the genotype distribution did not significantly depart from the Hardy-Weinberg equilibrium (chi-squared 0.0003). Patients diagnosed with T1DM before 30 years of age showed significantly more risk (A) alleles than the LADA group, both using the recessive and the dominant models, whereas T2DM showed fewer risk alleles, and a similar genotype distribution to controls. No significant differences were found between patients with LADA and those with T1DM diagnosed after the age of 30 using any of the models.

## Discussion

Our results show genetic differences between T1DM and LADA in all the analyzed genes (*HLA, PTPN22* and *INS*), but only in patients with T1DM diagnosed before 30 years of age. However, no significant differences were found between LADA and the group with T1DM diagnosed after the age of 30. When compared with T2DM, LADA patients had similar or higher frequencies of T1DM risk alleles.

The role of high risk alleles in the age of diagnosis of autoimmune diabetes has been previously studied by other authors, suggesting an earlier onset in those patients with the highest risk alleles (13), thus establishing a continuum from the pediatric age to adulthood. Still, environmental factors can alter this “continuum”, favoring earlier onset in those patients with lower genetic risk exposed to environmental triggers (12) such as viral infections, nutritional practices or increased BMI (14). In this context, LADA seems to be one of the lowest risk forms of autoimmune diabetes, presenting at a later age. Still, it is worth mentioning that LADA also shows a “spectrum” of presentation, with age of onset having been associated with the genetic risk (15) or GAD antibody titers (16). As Desai et al. phrased it, “supporting the hypothesis that autoimmune diabetes occurring in adults is an age-related extension of the pathophysiological process presenting as childhood-onset T1DM” (15).

To our knowledge, this is one of the first genetic studies to directly compare LADA and T1DM diagnosed after the age of 30. Indeed, previous genetic studies comparing LADA and T1DM are scarce overall. Other strengths of this study include the relatively large population and the adjustment for potential confounders (age and BMI).

As genetic analysis has become more accessible to medical and research centers around the world, the number of studies evaluating the genetic differences between the subtypes of DM has increased in recent years, providing novel insights into the pathogenic routes of the disease. In 2018, Cousminer et al (4) published the first Genome Wide Association Study (GWAS) in subjects with LADA (see **Table 5**), whom they compared not only to healthy controls, but also to patients diagnosed with T1DM and T2DM. They showed mixed genetic influence, sharing risk loci with both T1DM and T2DM. Compared to T1DM, the only significant differences were found in the *HLA* region, with LADA showing lower frequencies of high T1DM risk alleles. Compared to T2DM, LADA patients showed an increased frequency of T1DM risk alleles in *HLA*, *INS* (rs689), *PTPN22* (rs2476601) and *SHB3* (rs3184504). Similar results were published by Buzzetti et al. in 2007 (23), reporting higher frequencies of moderate and high risk class II HLA haplotypes in subjects diagnosed with LADA compared with patients diagnosed with T2DM.

**Table 5:** Studies assessing HLA, *PTPN22* and *INS* in LADA, type 1 and type 2 diabetes.

Author	Year	Type of article. Population	Subjects (Diagnosis (N))	HLA	*PTPN22* (rs2476601)*	*INS* (rs689)
Cervin et al. (17)	2008	Original. Sweden	T1DM (718), LADA (361), T2DM (1676) & healthy controls (1704)	Similar high-risk HLA in LADA vs T1DM. Increased protective HLA in LADA vs T1DM	Risk genotypes (CT/TT) increased in both T1DM and LADA vs controls	Risk genotype (AA) increased in T1DM and LADA vs controls
Pettersen et al. (16)	2010	Original. Norway	T1DM (120), LADA (126), T2DM (1090) & healthy controls (1503)	Risk haplotype decreased and protective haplotype increased in the following order: early T1D–late T1DM–high antiGAD LADA–low antiGAD LADA–T2DM–control	Associated with T1DM but not with LADA	Associated with T1DM but not with LADA
Andersen et al. (18)	2010	Original. Finland	T1DM>35y (257), T2DM & LADA (213)	Risk genotypes more frequent in T1DM vs LADA, and in LADA vs T2DM	Risk genotypes more frequent in T1DM vs LADA, and in LADA vs T2DM	Risk genotypes linked to T1DM. No difference in LADA vs T2DM
Weber et al. (19)	2010	Original. Czech Republic	T1DM>35y (41), LADA (61) & healthy controls (99)	High risk alleles (DRB1*03, 04) more frequent in T1DM vs LADA and vs controls	————————	————————
Kisand et al. (20)	2012	Original. Estonia	T1DM (154), T2DM (260), LADA (65) & healthy controls (229)	LADA associated with T1DM protective haplotypes	Increased risk of T1DM (T/T OR=7.2) vs controls. No effect found on LADA	Protective for T1DM (T/T phenotype, OR=0.06). No effect on LADA
Okruszko et al. (13)	2012	Original. Poland	T1DM (175), LADA (80) & healthy controls (151)	Frequency of high-risk genotypes decreased with age for both <T1DM and LADA. Protective genotypes increased with age	T/T genotype was more frequent in T1DM and LADA vs controls. Increased frequency with age in T1DM	————————
Dong et al. (21)	2014	Meta-analysis	6 studies: PTPN22: LADA (1088) & controls (4079)	————————	T allele increased risk of LADA vs controls (OR=1.52). Homozygous (T/T), OR=1.86; heterozygous (C/T), OR=1.52.	————————
Cousminer et al. (4)	2018	GWAS European	LADA (2634) & controls (5947) LADA (2454) & T1DM (968) LADA (2779) & T2DM (10396)	LADA showed lower frequencies of T1DM risk HLA alleles	Increased frequency in T1DM and LADA vs T2DM. No difference between T1DM and LADA.	Higher frequency in T1DM and LADA vs T2DM. No difference between T1DM and LADA.
Ramu et al. (22)	2019	Meta-analysis	16 studies. *PTPN22*: LADA (3187) & controls (7480) *INS:* LADA (4073) & controls (8307)	————————	T allele increased risk of LADA vs controls (OR=1.6). In homozygous (T/T) the risk increased (OR=2.67)	T allele protective for LADA (OR=0.6). In homozygous (T/T), OR=0.43; in heterozygous (A/T), OR=0.53
Buzzeti et al. (23)	2007	Original. Italy	T2DM (382) & LADA (191)	Higher frequency of moderate and high risk HLA class II haplotypes in LADA (22.5% and 8.4% respectively) compared with T2DM (11.2% and 1.2% respectively)	————————	————————

*Some authors mention the T allele to express the risk, and others mention the complementary nucleotide A. T is used throughout the table and text, for clarity’s sake. T1DM, type 1 diabetes; T2DM, type 2 diabetes.

In the present study, the patients diagnosed with T1DM overall also showed a higher frequency of the HLA risk haplotype DRB1*04-DQB1*03:02-DQA1*03:01 compared to LADA. Furthermore, the absence of risk alleles was more frequent in patients diagnosed with LADA than in patients diagnosed with T1DM, in agreement with Cousminer et al. (4). Indeed, the authors conclude that the leading genome-wide signals support the concept of LADA being a late-onset T1DM. Kisand et al. (20) also reported differences in *HLA* risk alleles between T1DM (mean age 22 years) and LADA in an Estonian population, i.e. an increased prevalence of T1DM *HLA* protective haplotypes in patients with LADA. This finding is in accordance with earlier studies suggesting that the increased frequency of protective HLA genotypes is a specific feature of patients with slowly-progressing T1DM. We did not find significant differences in genotypes when comparing LADA and T1DM with onset after 30 years of age, although given the low number of patients in the >30 years age group compared to the overall T1DM population we cannot rule out the existence of differences. Indeed, other authors have reported differences between these two groups (18, 24).

The presence of the T allele in rs2476601 in *PTPN22* has been reported to increase the risk of T1DM (20, 26) and LADA (17, 25) by some authors, though it is not a generalized finding in the latter (20). Studies focused on evaluating the differences between its frequency in patients with T1DM and patients with LADA are scarce. We found the risk variant more frequently in patients diagnosed with T1DM before de age of 30 years than in those with LADA, even though the result was statistically borderline (p=0.045). No differences were found on those diagnosed after the age of 30 (see **Table 4**). Authors have reported an increased presence of the risk (T) allele in both patients with T1DM and LADA when compared to healthy controls (17). Dong et al. reported a similar effect of the *PTPN22* gene (rs2476601) in their 2014 meta-analysis, when comparing patients diagnosed with LADA with healthy controls. They found an increased risk for LADA in carriers of the risk allele (21). Similarly, this T allele has also been shown to confer an increased risk of T1DM (5, 25–27).

On the other hand, Kisand et al. (20) reported no association between *PTPN22* genotypes and LADA susceptibility, but did find an increased risk for T1DM associated to rs2476601, when comparing both types of DM with controls. Interestingly, there was a 30-year difference in the mean age of diagnosis between the groups. In their 2019 meta-analysis, Ramu et al. (22) also compared subjects with LADA and healthy controls, and reported a significant association for the risk allele in rs2476601 with LADA in the codominant, dominant and recessive models.

Regarding the *INS* rs689 SNP, we also found a different genotypic distribution among our patients, with a reduced frequency of the (T) minor allele in patients diagnosed with T1DM when compared with LADA or T2DM, though no significant difference was seen between LADA and T1DM patients with an onset of >30 years (see **Table 4**). Kisand et al. (19) reported similar findings after comparing their patients with T1DM, T2DM and LADA. The T allele was associated with a reduced risk of T1DM, but was not associated with the risk of LADA. In their meta-analysis, Ramu et al. (22) also showed an inverse association between LADA and the presence of the T allele in the codominant, dominant and recessive models.

We acknowledge this study has some limitations. Even though our study is one of the largest published to date comparing genetic differences between T1DM and LADA, the relatively small sample size could limit our ability to detect minor differences, especially in the group with T1DM diagnosed after the age of 30 years. For unknown reasons, the number of male participants exceeds that of females. This may have influenced some of the results. Also, we limited our study to three of the main genes related to T1DM risk. More extensive studies assessing polygenic risk scores, would be interesting to perform.

To summarize, we present one of the few studies to directly compare differences in the genetic profile of patients with T1DM and LADA. We believe this differentiation is important because the underlying autoimmune process against the β-cell can have consequences for the prognosis, complications and treatment of patients (24). We report a similar T1DM risk allele distribution in patients with LADA compared to individuals with T1DM diagnosed after the age of 30, but less risk alleles than in those diagnosed before the age of 30 years. Our results suggest that, from a genetic point of view, LADA holds genetic similarities to late-onset forms of T1DM. Our study supports the importance of genetic studies in the characterization of LADA, but also of comparing the study group with appropriate controls, although the confidence in the results is limited by sample size.

## Data availability statement

The original contributions presented in the study are included in the article/supplementary files. Further inquiries can be directed to the corresponding authors.

## Ethics statement

The studies involving human participants were reviewed and approved by Ethics Committees of Hospital de la Santa Creu i Sant Pau, Barcelona and Hospital Universitari Arnau de Vilanova, Lleida. The patients/participants provided their written informed consent to participate in this study.

## Author contributions

MH and DM were responsible for study conception and design, RF, EP, AE, EC and MH participated in data acquisition. RF and EP performed data analysis. AM and YN-M drafted the first version of the manuscript. MH, DM, AM and YN-M made subsequent revisions to the manuscript. All authors revised and approved the final version of the paper.

## Funding

This project was supported by a grant from Instituto de Salud Carlos III, Spain (Project FIS 061104).

## Acknowledgments

The authors thank Associació Catalana de Diabetis (ACD) for providing the samples and clinical information of the patients included in the register. We acknowledge Joan Verdaguer, from the University of Lleida (UdL), for logistic support with the genetic studies.

## Conflict of interest

The authors declare that the research was conducted in the absence of any commercial or financial relationships that could be construed as a potential conflict of interest.

## Publisher’s note

All claims expressed in this article are solely those of the authors and do not necessarily represent those of their affiliated organizations, or those of the publisher, the editors and the reviewers. Any product that may be evaluated in this article, or claim that may be made by its manufacturer, is not guaranteed or endorsed by the publisher.
